# Metabolic Power Requirement of Change of Direction Speed in Young Soccer Players: Not All Is What It Seems

**DOI:** 10.1371/journal.pone.0149839

**Published:** 2016-03-01

**Authors:** Karim Hader, Alberto Mendez-Villanueva, Dino Palazzi, Saïd Ahmaidi, Martin Buchheit

**Affiliations:** 1 Laboratory of Exercise Physiology and Rehabilitation, EA 3300, Faculty of Sport Sciences, University of Picardie, Jules Verne, 80025 Amiens, France; 2 National Sports Medicine Programme, Excellence in Football Project, Aspetar-Orthopaedic and Sports Medicine Hospital, Doha, Qatar; 3 Sport Science Department, Aspire Academy, Doha, Qatar; 4 Sport Science Department, Myorobie Association, Montvalezan, France; 5 Performance Department, Paris Saint Germain Football Club, Saint-Germain-en-Laye, France; 6 Institute of Sport, Exercise and Active Living, College of Sport and Exercise Science, Victoria University, Melbourne, Australia; West Virginia University, UNITED STATES

## Abstract

**Purpose:**

The aims of this study were to 1) compare the metabolic power demand of straight-line and change of direction (COD) sprints including 45° or 90°-turns, and 2) examine the relation between estimated metabolic demands and muscular activity throughout the 3 phases of COD-sprints.

**Methods:**

Twelve highly-trained soccer players performed one 25-m and three 20-m sprints, either in straight-line or with one 45°- or 90°-COD. Sprints were monitored with 2 synchronized 100-Hz laser guns to assess players’ velocities before, during and after the COD. Acceleration and deceleration were derived from changes in speed over time. Metabolic power was estimated based on di Prampero’s approach (2005). Electromyography amplitude (RMS) of 2 lower limb muscles was measured. The expected energy expenditure during time-adjusted straight-line sprints (matching COD sprints time) was also calculated.

**Results:**

Locomotor-dependant metabolic demand was *largely* lower with COD (90°, 142.1±13.5 J.kg^-1^) compared with time-adjusted (effect size, ES = -3.0; 193.2±18.6 J.kg^-1^) and non-adjusted straight-line sprints (ES = -1.7; 168.4±15.3 J.kg^-1^). Metabolic power requirement was angle-dependent, *moderately* lower for 90°-COD vs. 45°-COD sprint (ES = -1.0; 149.5±10.4 J.kg^-1^). Conversely, the RMS was *slightly*- (45°, ES = +0.5; +2.1%, 90% confidence limits (±3.6) for vastus lateralis muscle (VL)) to-*largely* (90°, ES = +1.6; +6.1 (3.3%) for VL) greater for COD-sprints. Metabolic power/RMS ratio was 2 to 4 times lower during deceleration than acceleration phases.

**Conclusion:**

Present results show that COD-sprints are largely less metabolically demanding than linear sprints. This may be related to the very low metabolic demand associated with the deceleration phase during COD-sprints that may not be compensated by the increased requirement of the reacceleration phase. These results also highlight the dissociation between metabolic and muscle activity demands during COD-sprints, which questions the use of metabolic power as a single measure of running load in soccer.

## Introduction

In team sports (e.g., soccer, basket-ball, hand-ball), the ability to quickly accelerate/decelerate during sprints with or without a change of direction (COD) is decisive for game-deciding situations (e.g., winning a ball, creating and stopping goal scoring opportunities) [1–3]. Professional soccer players can perform on average more than 8 CODs per minute during a match [4] and about 3 fold more high accelerations (> 2.5 m.s^-2^) than sprints [5]. In addition, the ability to change of direction while running has been recognized as an important factor for a successful participation in team sports [6]. The acceleration-deceleration dynamics associated with repeated CODs require high levels of mechanical (e.g., eccentric contractions) and metabolic load [7], which may be reflected by increases in markers of muscular damage following soccer training [8] and matches [9]. The exact mechanical and metabolic responses to COD speed remain however unclear, and the influence of COD angle and speed has not been clearly established. A better understanding of the energy demands of un-orthodox movement patterns [2] which characterize COD speed may be useful to assess the actual energy requirements of training and match situations.

Empirical and scientific evidences suggest that running with COD increases the energy demands of human locomotion. Compared with straight-line runs, greater blood lactate concentration ([La]_b_), heart rate (HR) [10, 11] and oxygen uptake (VO_2_) [10] have been reported during both supramaximal [10, 11] and submaximal [12] running exercises. In these studies however, speed or distance was not adjusted for the time lost during the turns, so that the respective effect of COD *per se* could not be properly examined. In a recent study, Hatamoto et al. [13] estimated the energy cost of a single 180°-COD using repeated runs with different turns frequency (i.e., different frequency accumulation method). Results showed that the energy cost increased with running velocity, but only very low intensities were examined (i.e., < 9 km.h^-1^); the actual cost of COD at higher running speed as during training and matches remains unknown. Another alternative to isolate the metabolic effect of CODs *per se* is to use runs over time-adjusted distances, i.e., adjusted COD running distances matched for straight-line sprint time [14, 15]. Compared with straight-line runs, COD-adjusted runs elicited equivalent VO_2_ and variable [La]_b_ responses: [La]_b_ were lower during repeated sprints with COD [12] but greater during high-intensity intermittent runs [15]. These latter measures were however limited to systemic/whole-body responses. The direct limitation of this approach is that the estimated energy demands can be contaminated by the additional requirements of the upper-body limbs [10], and that exclusively locomotor-related demands can’t be isolated.

To assess the locomotor-related energy demands in the field, di Prampero et al. [16] introduced a new approach based on the estimated energy cost of accelerated and decelerated running. While this approach has been used in three studies to report the overall locomotor-related metabolic demands during soccer games and during submaximal intermittent shuttle runs [7, 17, 18], the soccer-specific (i.e., between 0 and 90°-COD) demands of COD-sprints is still unknown. Additionally, the respective contribution of the deceleration, turning and re-acceleration phases to the overall COD energy requirement hasn’t been examined. Practically, the methods generally used to assess locomotor patterns in the field during training and matches may have limitations when it comes to the investigation of COD speed. For instance, global positioning systems (GPS) or local position measurements are commonly-used but may have limited validity and reliability for such short and intense movement patterns [19–21]. The use of laser guns, which present acceptable reliability and validity to assess changes in velocities [22], may offer an alternative to practitioners willing to assess the different kinematic phases and metabolic requirement of COD speed.

In addition to the aforementioned metabolic responses, CODs affect substantially lower limb muscle activity as assessed via electromyography (EMG). Compared with straight-line runs, EMG activity has been reported to increase during runs with COD [23, 24]. This increase may be related to the increased external load placed on the knee joint [23] and the need for applying high lateral forces on the ground [25]. However, very little is known about the changes in lower limb muscle activity during the different phases of the CODs (i.e., acceleration, deceleration, turn). Moreover, how these potential changes in EMG activity relate to changes in metabolic requirement during the different phases of COD speed is also unknown. Finally, since performance [6, 26], physiological and muscular activity [14, 15] responses during COD speed are likely COD angle-dependent, the locomotor-related metabolic demands and their relationship with muscular activity may also be COD-angle dependent. However, this has still to be examined.

In an attempt to describe the detailed kinematics and metabolic demands of the different COD phases in the field, we have recently developed a new timing system combining two laser guns, which allows the continuous tracking of the players before, during and after the COD [22]. This system allows the monitoring of different center of mass (COM)-related kinematic variables during COD speed [22], with reasonable levels of reproducibility (small-to-moderate standardized coefficients of variation (CVs) [22]. The aims of the present study were therefore to examine 1) the metabolic power demands of field-based straight-line and COD sprints including either 45° or 90°-turns, while accounting or not for the time lost when changing direction, 2) the relation between estimated metabolic demands and lower limb muscles activity and, 3) an eventual angle-dependence of metabolic and lower limb muscles activity demands during COD-sprints.

## Materials and Methods

### Participants

Twelve highly-trained young soccer players (age: 16.5 ± 0.4 yr, age from estimated peak height velocity [27]: 2.1 ± 0.6 years old, height: 170.3 ± 6.4 cm, body mass 60.0 ± 6.3 kg, sum of 7 skinfolds: 45.6 ± 16.0, 10-m sprint time: 1.76 ± 0.05 s and maximal sprinting speed: 29.9 ± 1.2 km.h-1) from an elite academy were involved. Anthropometric and performance data were collected as previously described. All the players participated on average in ~14 hours of combined soccer-specific training and competitive play per week (6–8 soccer training sessions, 1 strength training session, 1–2 conditioning sessions, 1 domestic game per week and 2 international club games every 3 weeks). All players had a minimum of 3 years prior soccer-specific training and were well familiar with the testing procedures. Written informed consent was obtained from the players and their parents. The study was approved by the Anti-Doping Lab Qatar (ADLQ) Ethics Committee and conformed to the recommendations of the Declaration of Helsinki.

### Experimental overview

**Fig 1:**
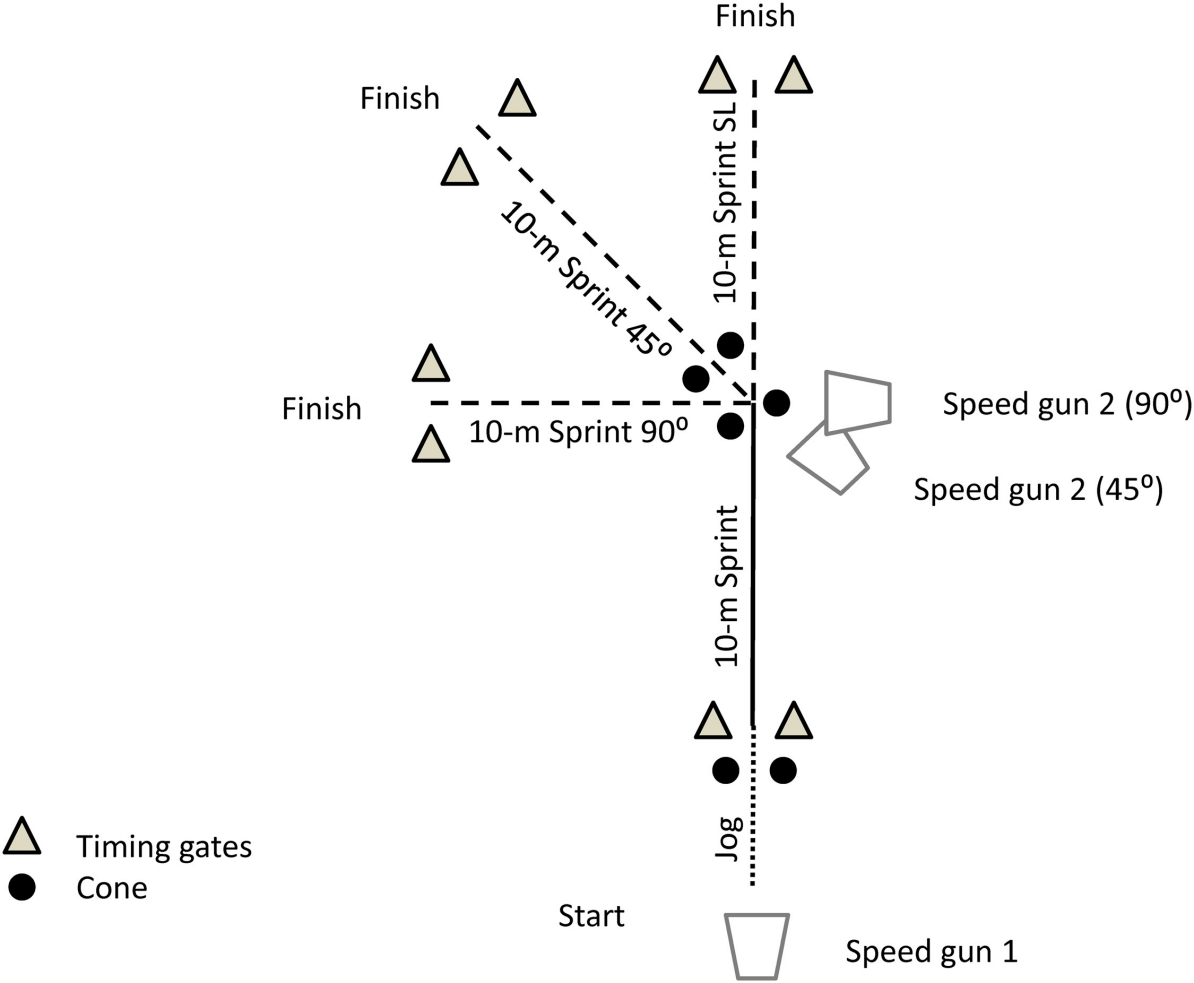
Experimental set up with the new timing methodology combining two speed guns synchronized. See methods for details.

Following a 15-min standardized warm-up, including eight consecutive COD-runs with progressive increased speed for familiarization, players randomly performed two 40-m sprints in straight-line (SL) with 10-m splits, two 20-m sprints with one left 45°-COD, two 20-m sprints with one left 90°-COD after 10 m, and two 25-m sprints with one left 90°-COD after 15 m (90°_25_). The angles of 45° and 90° were chosen since the majority of COD-runs in soccer matches occur within a range of 0 to 90° [4]. The use of a single COD during the sprints was chosen to determine the energy demand per COD and also for specificity with regard to soccer practice during matches [28]. As a part of the academy performance screening (i.e., three times per year), players’ anthropometric measures and maximum sprinting speed [29] were available and then, included as possible determinants to COD-sprint performance. In addition, all the players were familiarized with this type of COD-sprint while being routinely tested during the academy performance screening on a similar 90°-COD sprint. Players were required to initiate the left turn with a strong impulse of their right foot, positioned in the centre of the running course, at the level of the turn. A posteriori, it appeared on videotape that all the players performed naturally the 90°-COD sprints as requested (i.e., strong right foot impulse to initiate the turn). Players’ dominant leg (i.e., the kicking leg) was the right one for all. In the present study, all players turned on the left during the COD-sprints. Whether different responses could have been observed with a right turn could not be examined in the present study, which is a limitation. However, Castillo-Rodriguez et al. [30] observed that amateur players kicking with their right foot were very likely to present a greater COD-sprint performance on the left side. All players turned largely faster to the left side than the right side (i.e., effect size = 1.8). There was a 2–3 min passive recovery period between each sprint. To increase ecological validity, players commenced each sprint from a jogging start (2 m.s^-1^, controlled with a metronome) over 10 m, and were instructed to initiate their sprint when reaching a cone placed 1 m from the starting line (Fig 1). Tests were performed with soccer boots on an outdoor (temperature 39.5 ± 1.5°C and relative humidity 18 ± 2.6%) grass soccer pitch.

To account for the time lost while changing direction, the distance for straight-line sprints was individually adjusted [15] using the ratio between the straight-line and COD-sprints as follows:

$$Adjusted\;straight\;line\;distance\;\left(m\right)\;=\frac{CODsprint\;time\;\left(s\right)\cdot CODsprint\;distance}{Straight\;sprint\;time\;\left(s\right)}$$ (1)

Accordingly to Eq (1), we calculated adjusted straight-line distances corresponding to 20-m 45°-COD sprints, 20-m and 25-m 90°-COD sprints.

### Estimated metabolic demands using di Prampero’s approach

Di Prampero et al. [16] suggested that, accelerated running on flat terrain, as a first approximation, is biomechanically equivalent to running uphill at constant speed, up an ‘‘equivalent slope” (ES) dictated by the forward acceleration [16]. Minetti et al. [31] have shown a relationship between the energy cost of constant-speed running and inclination of the terrain over a wide range of up- or down-slopes. Based on this latter study, di Prampero et al. [16] proposed an equation to estimate the instantaneous energy cost of accelerated running as follows:

$$C=\left(155.4\cdot ES^{5}–30.4\cdot ES^{4}–43.3\cdot ES^{3}+46.3\cdot ES^{2}+19.5\cdot ES+3.6\right)\cdot EM\cdot KT`$$ (2)

in which *C* is the energy cost of accelerated running on the specific terrain (in J·kg^-1^·m^-1^) calculated with di Prampero’s approach, *ES* is the equivalent slope: ES = tan(90-arc_tan_ g/a_f_); g = acceleration due to gravity; a_f_ = forward acceleration; 3.6 is the energy cost of running on flat terrain at constant speed; *EM* is the equivalent body mass: EM = (a_f_^2^/g^2^ +1)^0.5^; and *KT* is a terrain constant (KT = 1.29, [7]) to take into account the fact that running on a football field is approximately 30% more costly than running on compact homogeneous terrain.

The metabolic power (P; W·kg^-1^) was then calculated by multiplying C (J·kg^-1^·m^-1^) with the estimated (i.e., obtained per meter) speed (v; m·s^-1^) as follows:

P = C·v (3)

Finally, the total estimated energy expenditure (EEE; J·kg^-1^) was determined as the sum of instantaneous EEE obtained for each meter of each sprint.

### Center of mass-related kinematic measures

Sprints were simultaneously monitored with timing gates (Brower Timing System, Draper, UT, USA, 1 ms resolution) and two cabled-synchronized 100-Hz laser guns (Laveg LDM100, Jenoptik, Germany, Fig 1). A custom-developed spreadsheet gathered both data files and calculated the whole player’s running profile before, during and after the COD [22]. Individual laser measurements show very good validity (average velocity error of ~2% and reproducibility (coefficient of variation, CV: 1–3%) when assessing linear speed [32]. For the purpose of the present study, the reliability of metabolic power during COD speed was also assessed (Table 1). The lower ICC for the 45° trial may be related to the fact that compared with the straight-line or 90°-COD sprints, players could adopt slightly different running patterns when passing the cones. While the players had learnt in the academy to clearly position their right foot to initiate the left turn with a strong impulse on the ground during the 90°-COD sprint, turning at 45° at high speed could be achieved using either the right or the left foot. This may be associated with greater variations in the actual running path and/or body position, which may have increased the possible time differences between the trials [22].

**Table 1:** Reliability of some metabolic power variables collected with the new timing methodology and timing gates during sprints with and without change of direction.

		Difference (%)	CV (%)	ICC
**Mean metabolic power (W.kg**^**-1**^**)**	Straight-line	-0.2 (-4.7;4.5)	4.0 (2.7;8.6)#	0.95 (0.76;0.99)***
	45°	0.2 (-1.0;1.1)	6.4 (4.5;11.2)##	0.47 (0.02;0.79)
	90°	-0.1 (-6.2;6.4)	8.0 (5.8;13.4)##	0.80 (0.44;0.93)**
**Peak metabolic power (W.kg**^**-1**^**)**	Straight-line	-1.2 (-8.6;7.4)	12.5 (8.9;21.3)##	0.61 (0.16;0.90)*
	45°	1.1 (-6.8;9.6)	10.4 (7.5;17.7)##	0.36 (-0.20;0.74)
	90°	0.7 (-3.5;5.1)	5.4 (3.9;9.0)#	0.70 (0.28;0.90)*

Between-trial difference (90% confidence limits), typical error expressed as a coefficient of variation (CV, 90% confidence limits) and intraclass correlation coefficient (ICC, 90% confidence limits). The number of ‘#’ symbols stands for small, moderate, large and very large standardized difference and CV, respectively. For ICC values, the number of ‘*’ symbols refers to moderate, large and very large magnitudes, respectively.

### Electromyography measurement

Electromyography (EMG) data were collected from the dominant leg (i.e., the leg used to both kick and turn for the COD), using a sixteen channel Trigno Wireless EMG system (Delsys INC, Boston, USA). The contracted muscle belly of the vastus lateralis (VL) and biceps femoris (BF) were identified. Before placing the electrodes in accordance with the Surface EMG for Non-invasive Assessment of Muscles recommendations (SENIAM) [33]. The overlying skin was carefully prepared. The hair was shaved, and the skin was lightly abraded to remove the outer layer of epidermal cells and thoroughly cleansed with alcohol to reduce the skin—electrode interface impedance. Trigno wireless EMG sensors (4 silver bars contact) were carefully taped to the belly of each muscle, parallel to the muscle fibers, using hypoallergenic adhesive tape and cotton wool swabs to minimize sweat induced interference. Signals were sampled at 1000 Hz, amplified (1000×) and band-pass filtered (20–450 Hz). Data were imported from the Trigno base station and saved for offline analysis with Spike 2 version 5 (Cambridge Electronics Design, Cambridge, UK). The data were smoothed using route mean squared analysis (RMS), which was calculated for a 50-ms window. EMG data (μV) were calculated for each step (active contraction). Onset and offset of muscle activity were determined as a deviation greater than two standard deviations from the mean of three 50-ms windows of inactivity. The fastest 20-m straight-line sprint was also analysed by isolating peak amplitude contractions from the middle of the sprint. The resultant mean amplitudes were averaged and used for normalization, i.e., the EMG data from COD sprints were expressed as a percentage of the EMG measured during the fastest straight-line sprint [34]. Branch et al. [35] have shown that normalizing EMG to a functional task reduced inter-subject variability compared with normalizing to a maximum voluntary contraction. This approach has been used in several studies [23, 34, 36, 37] to normalize EMG signals during dynamic COD tasks.

### Data treatment

Raw (position) data from the first laser gun was zeroed at the starting line, while the second one was zeroed at the COD point. Velocity data was obtained by derivation and then processed using a 4^th^ order low-pass Butterworth digital filter with a cut-off frequency of 0.6 Hz (selected after several trials judged by visual inspection). Then, both speed curves were merged into a unique curve using the first laser readings at the beginning, the second one at the end; the merged interval (COD) was estimated by interpolation of both readings. Finally, data were resampled to provide an estimate of speed at each meter throughout the entire runs. Acceleration and deceleration were derived from meter-to-meter changes in speed over time. Metabolic power and estimated energy expenditure were estimated based on di Prampero’s approach [16]. Meter-to-meter RMS data were estimated by interpolation between each burst of muscle activity.

### Statistical analysis

Data in text, tables and figures are presented as mean with standard deviations and 90% confidence intervals/limits (CI/CL). All data were first log-transformed to reduce bias arising from non-uniformity error. The typical error of measurement, expressed as a coefficient of variation (CV, in % and standardized units) and the intraclass coefficient correlation (ICC) were used as measures of reliability [38].

Between-sprints standardized differences in the different running variables were also calculated, using pooled standard deviations. Uncertainty in the differences was expressed as 90% CL and as probabilities that the true effect was substantially greater or smaller than the smaller practical difference (between-subjects SD/5). These probabilities were used to make a qualitative probabilistic mechanistic inference about the true effect. The scale was as follows: 25–75%, possible; 75–95%, likely; 95–99%, very likely; >99%, almost certain. Threshold values for standardized differences were >0.2 (small), >0.6 (moderate), >1.2 (large) and very large (>2). The magnitude of the ICC was assessed using the following thresholds: >0.99, extremely high; 0.99–0.90, very high; 0.90–0.75, high; 0.75–0.50, moderate; 0.50–0.20, low; <0.20, very low [38]. Finally, the following criteria were adopted to interpret the magnitude of the correlation: ≤0.1, trivial; >0.1–0.3, small; >0.3–0.5, moderate; >0.5–0.7, large; >0.7–0.9, very large; and >0.9–1.0, almost perfect [39]. If the 90% CI overlapped small positive and negative values, the magnitude was deemed unclear; otherwise that magnitude was deemed to be the observed magnitude [38].

## Results

The level of reliability of metabolic power related variables was ranged from small to moderate CVs (Table 1).

The COD-time adjusted straight-line distances corresponding to 45°-, 90°- and 90°_25_-COD sprints are shown in Table 2.

**Table 2:** Non-adjusted and change of direction-time-adjusted straight-line distances.

	Distances (m)	Sprint time (s)	COD-time adjusted SL-distance (m)
**SL**	20	2.89 ± 0.13	
**45°**	20	3.30 ± 0.16 ****4	22.1 ± 1.1**ǂǂǂǂ**4
**90°**	20	3.70 ± 0.16 ****4††††4	25.1 ± 1.3 **ǂǂǂǂ**4
**90°**_**25**_	25	4.24 ± 0.18 ****4††††4	30.4 ± 1.6 **ǂǂǂǂ**4

COD: change of direction; SL: straight-line; COD-time adjusted straight-line distance: adjusted (i.e., extended) straight-line running distances matched for COD-sprint time.

The number of ‘*’ and ‘†’ refers to possible, likely, very likely and almost certain difference versus straight-line and 45°-COD sprint times, respectively. The number of ǂ refers to possible, likely, very likely and almost certain difference versus non-adjusted distances. The associated number refers to the magnitude of the difference, with 1 standing for small, 2 for moderate, 3 for large and 4 for very large magnitude.

**Fig 2:**
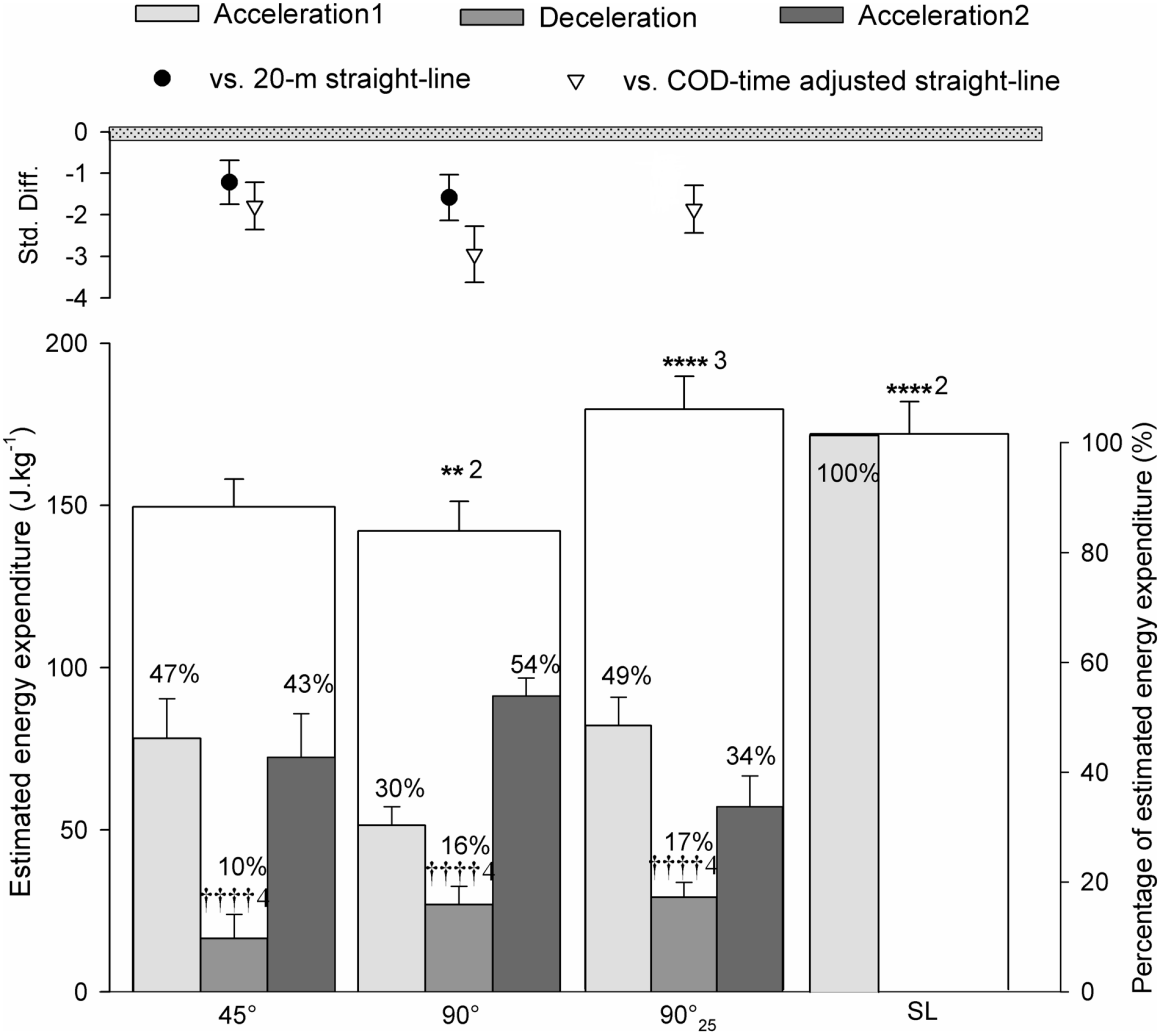
Estimated energy expenditure of sprints with (45° or 90°) or without (i.e., straight-line, SL) one change of direction (COD). 90°_25_: 25-m sprint with one 90°-COD. The upper panel represents the standardized difference (Std Diff) between COD- and SL sprints. Since 90°_25_ vs. 20-m SL sprints could not be properly compared (i.e., differences in both running time and distance), their standardized difference (black circle) was not provided. The number of ‘*’ and ‘†’ refers to possible, likely, very likely and almost certain between-sprints differences versus the 45°-COD sprint trial, and within-sprint differences vs. the acceleration phases, respectively. The associated number refers to the magnitude of the difference, with 1 standing for small, 2 for moderate, 3 for large and 4 for very large magnitude.

The EEE during the 20-m straight-line, 45°, 90° and 90°_25_-sprints is shown in Fig 2 and EEE of time-adjusted straight-line sprints were calculated (i.e., 178.9 ± 15.5 J.kg^-1^, 193.2 ± 18.6 J.kg^-1^ and 217.8 ± 15.5 J.kg^-1^ during straight-line adjusted for 45°- and 90°- and 90°_25_-sprint times respectively). The EEE of the 25-m straight-line sprints was also determined (i.e., 193.5 ± 15.3 J.kg^-1^). The EEE of COD-sprints were almost certainly lower compared with straight-line and even most likely lower compared with adjusted straight-line trials (Fig 2). EEE was also angle-dependent, almost certainly lower with 90°-COD than 45°-COD (Fig 2). For all COD-sprints, the estimated energy expenditure was almost certainly lower during the deceleration phases compared with the acceleration phases (Fig 2).

**Fig 3:**
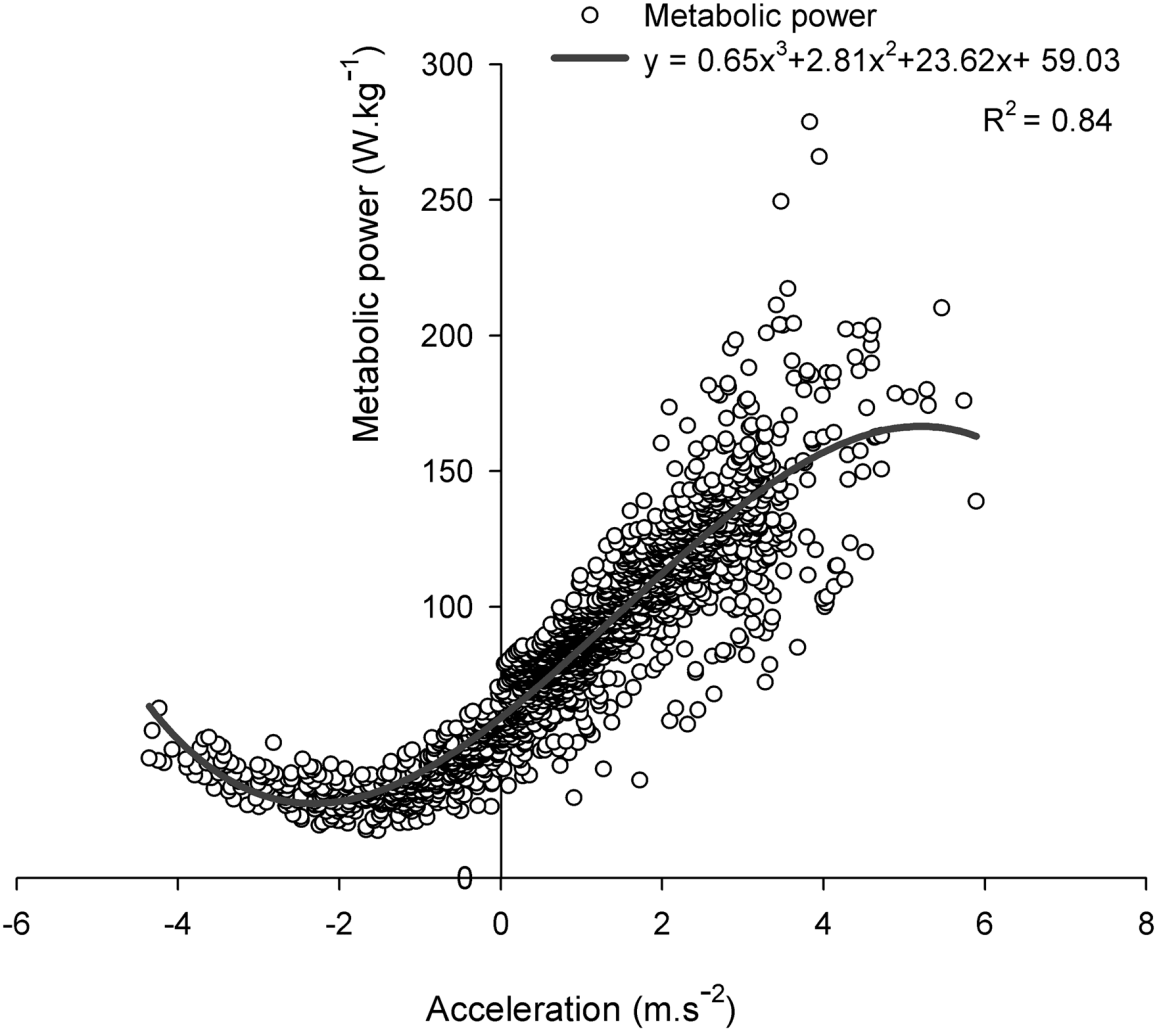
Relationship between acceleration and metabolic power.

The relationship between metabolic power and acceleration/deceleration could be represented by a cubic function with a minimum value of metabolic power (i.e., 8.03 ± 2.85 W.kg^-1^) associated with a deceleration of -2.26 ± 0.18 m.s^-2^ (Fig 3) and an energy cost of 2.60 ± 0.22 J.kg^-1^.m^-1^. Elevated decelerations (i.e., < -2 m.s^-2^, [7]) were observed at the 8^th^ (-2.34 ± 0.85 m.s^-2^), 9^th^ (-2.39 ± 0.82 m.s^-2^) and 10^th^ (-2.12 ± 0.71 m.s^-2^) meter of 90°-COD sprints and at the 12^th^ (-2.14 ± 0.93 m.s^-2^), 13^th^ (-2.39 ± 1.05 m.s^-2^), 14^th^ (-2.55 ± 1.26 m.s^-2^) and 15^th^ (-2.32 ± 1.40 m.s^-2^) meter of 90°_25_-COD sprints.

**Fig 4:**
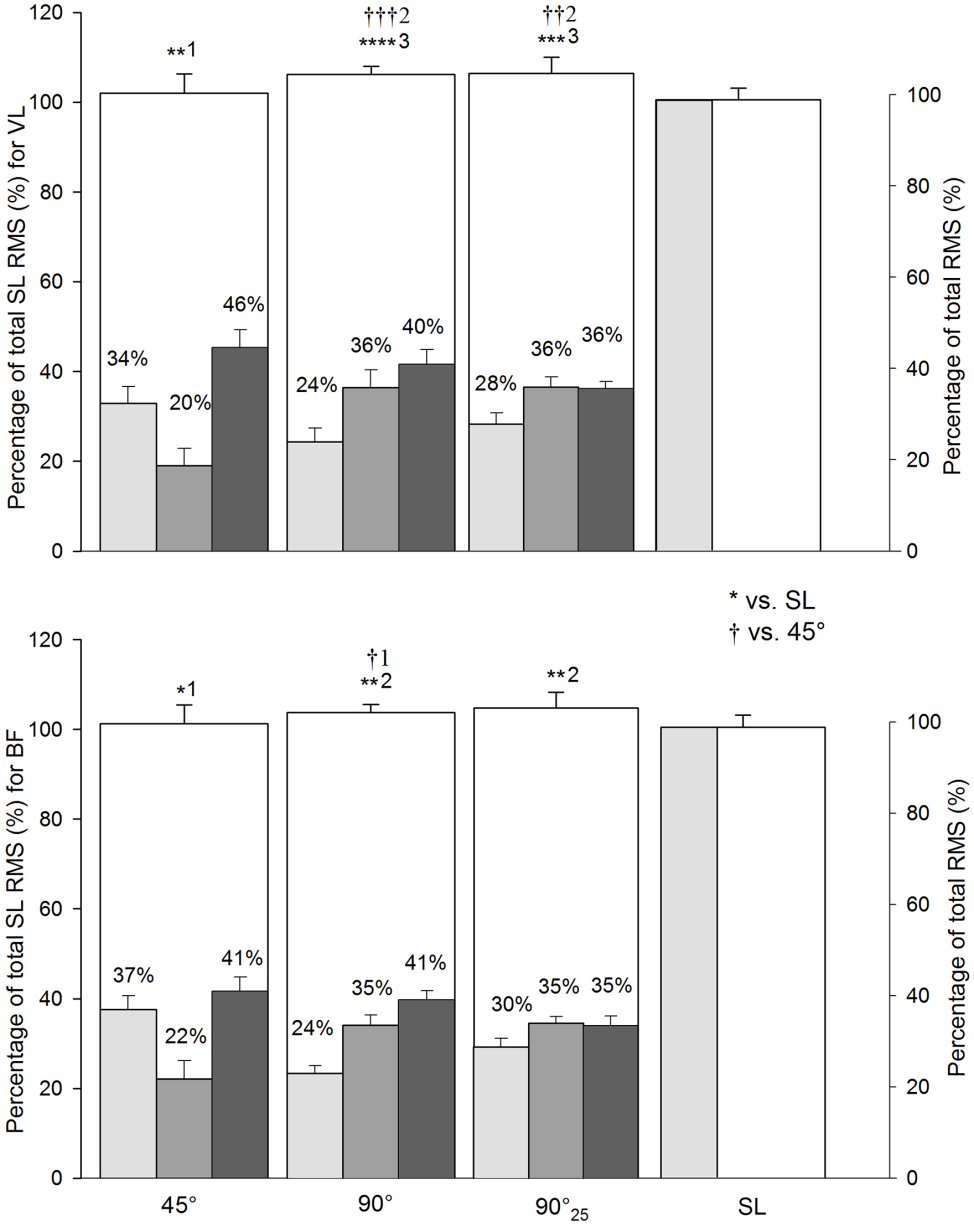
Electromyography amplitude (RMS) of 2 muscles during sprints with (45° or 90°) or without (i.e., straight-line, SL) one change of direction (COD). The upper panel concerns the vastus lateralis muscle and the lower panel, the biceps femoris muscle. 90°_25_: 25-m sprint with one 90°-COD. The number of ‘*’ and ‘†’ refers to possible, likely, very likely and almost certain difference versus straight-line and 45°-COD sprints, respectively. The associated number refers to the magnitude of the difference, with 1 standing for small, 2 for moderate, 3 for large and 4 for very large magnitude.

**Fig 5:**
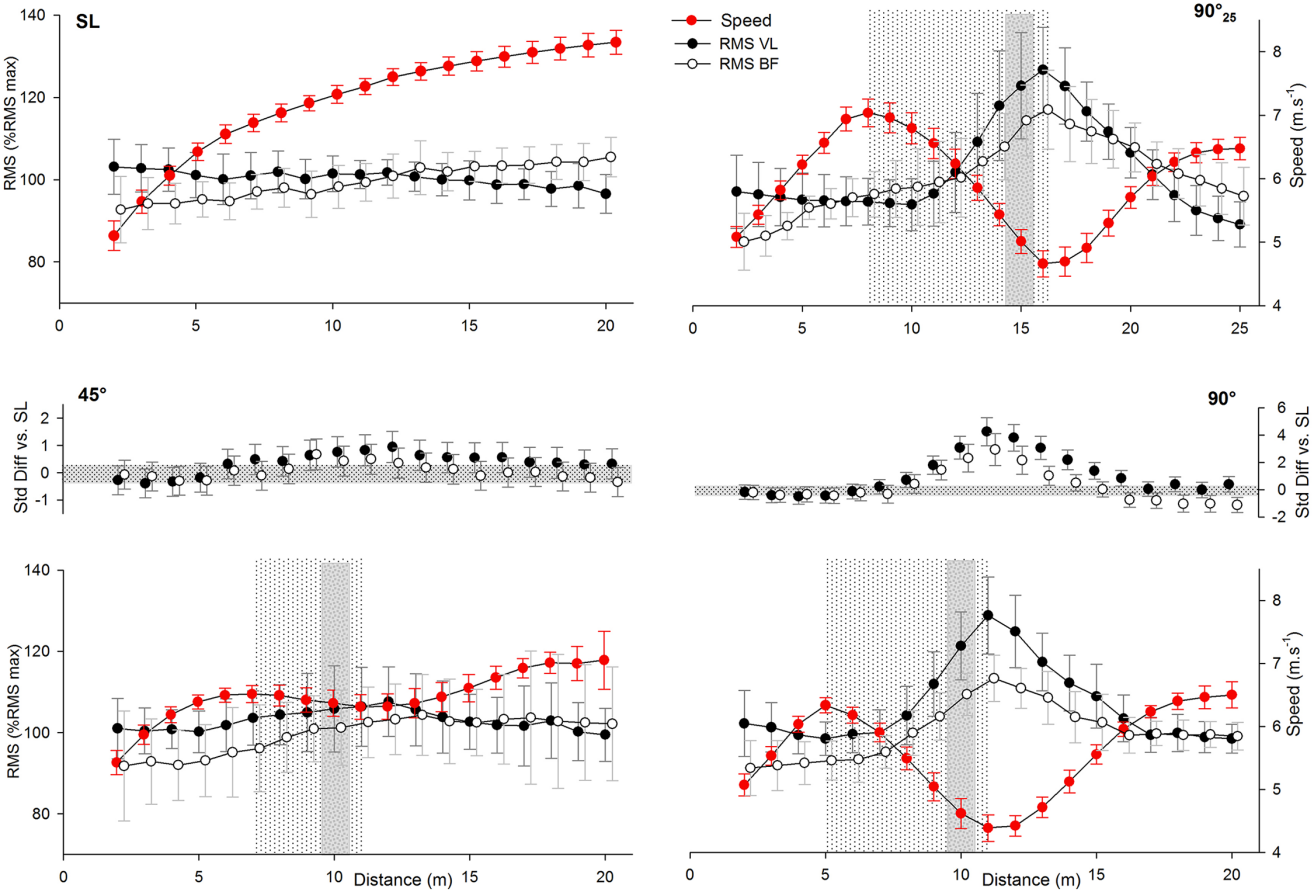
Electromyography amplitude (RMS) of vastus lateralis and biceps femoris muscles and speed profiles during sprints with (45° or 90°) or without (i.e., straight-line, SL) one change of direction (COD). 90°_25_: 25-m sprint with one 90°-COD. The medial panel represents the standardized difference (Std Diff) of RMS between COD- and SL sprints. The number of ‘*’ and ‘†’ refers to possible, likely, very likely and almost certain difference versus straight-line and 45°-COD sprints, respectively.

In average, VL and BF RMS for all COD sprints were possibly (i.e., 45°) and likely-to-almost certainly (i.e., 90° and 90°_25_) greater compared with straight-line (Fig 4). The EMG amplitude of both muscles were possibly (i.e., BF) -to-very likely (i.e., VL) greater during 90° than 45°-COD (Fig 4). The speed and RMS (VL and BF) profiles (i.e., per meter) during the straight-line, 45°, 90° and 90°_25_ sprints are shown in Fig 5. Compared with straight-line, EMG activity was possibly-to-likely greater during 45°-COD and almost certainly greater 90°-COD sprints between 8-m and 15-m (Fig 5).

The EMG amplitude during acceleration/deceleration phases was also angle-dependent; while VL and BF RMS were almost certainly greater during acceleration than deceleration with 90°-COD, they were almost certainly lower during 45°-COD sprints (Fig 5).

**Fig 6:**
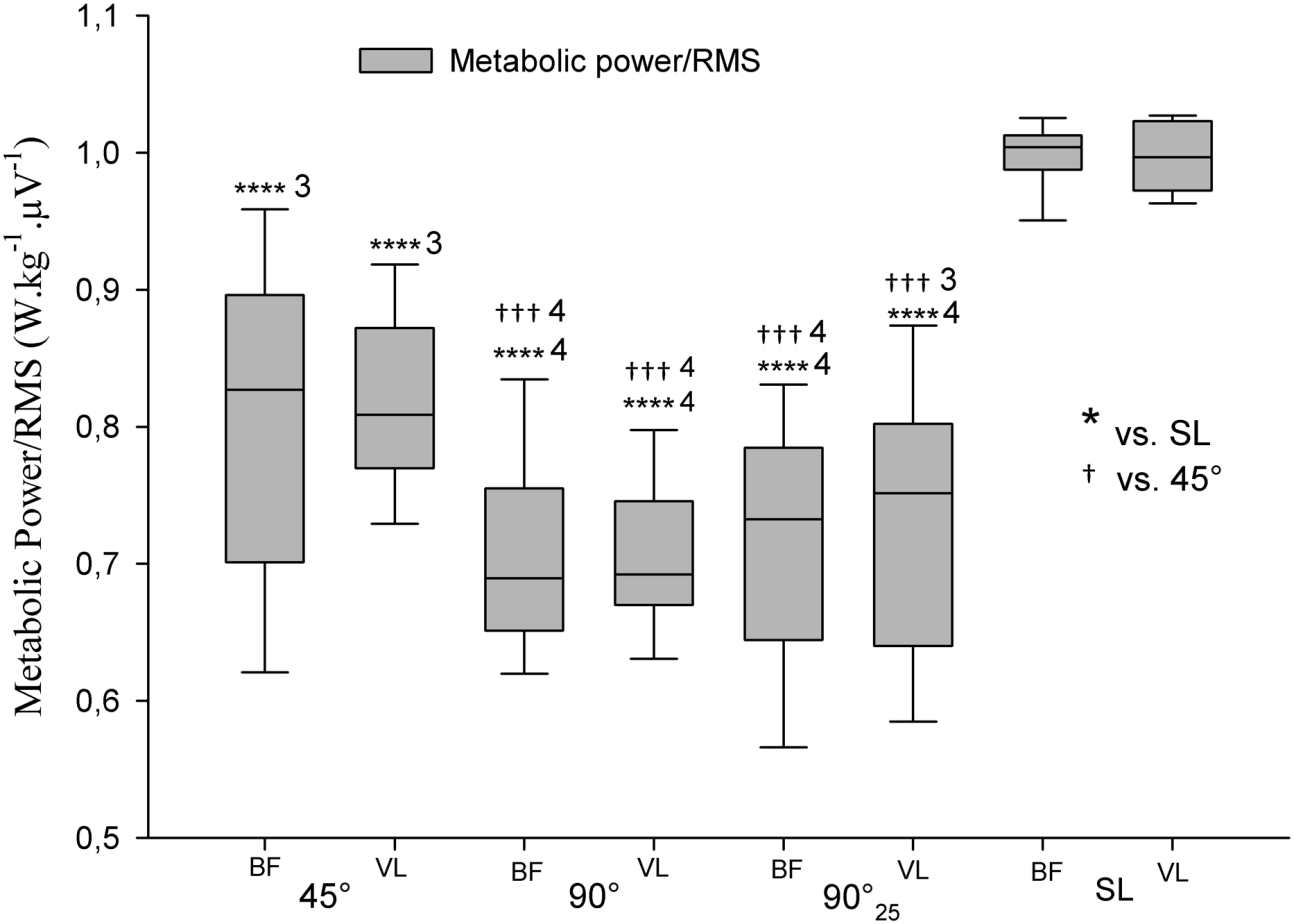
Metabolic power/electromyography amplitude (RMS) ratio of sprints with (45° or 90°) or without (i.e., straight-line (SL)) one change of direction (COD). 90°_25_: 25-m sprint with one 90°-COD; BF: biceps femoris; VL: vastus lateralis. The number of ‘*’ and ‘†’ refers to possible, likely, very likely and almost certain difference versus straight-line and 45°-COD sprints, respectively. The associated numbers represent the magnitude of the standardized difference, with 1 standing for small, 2 for moderate, 3 for large and 4 for very large magnitude.

**Fig 7:**
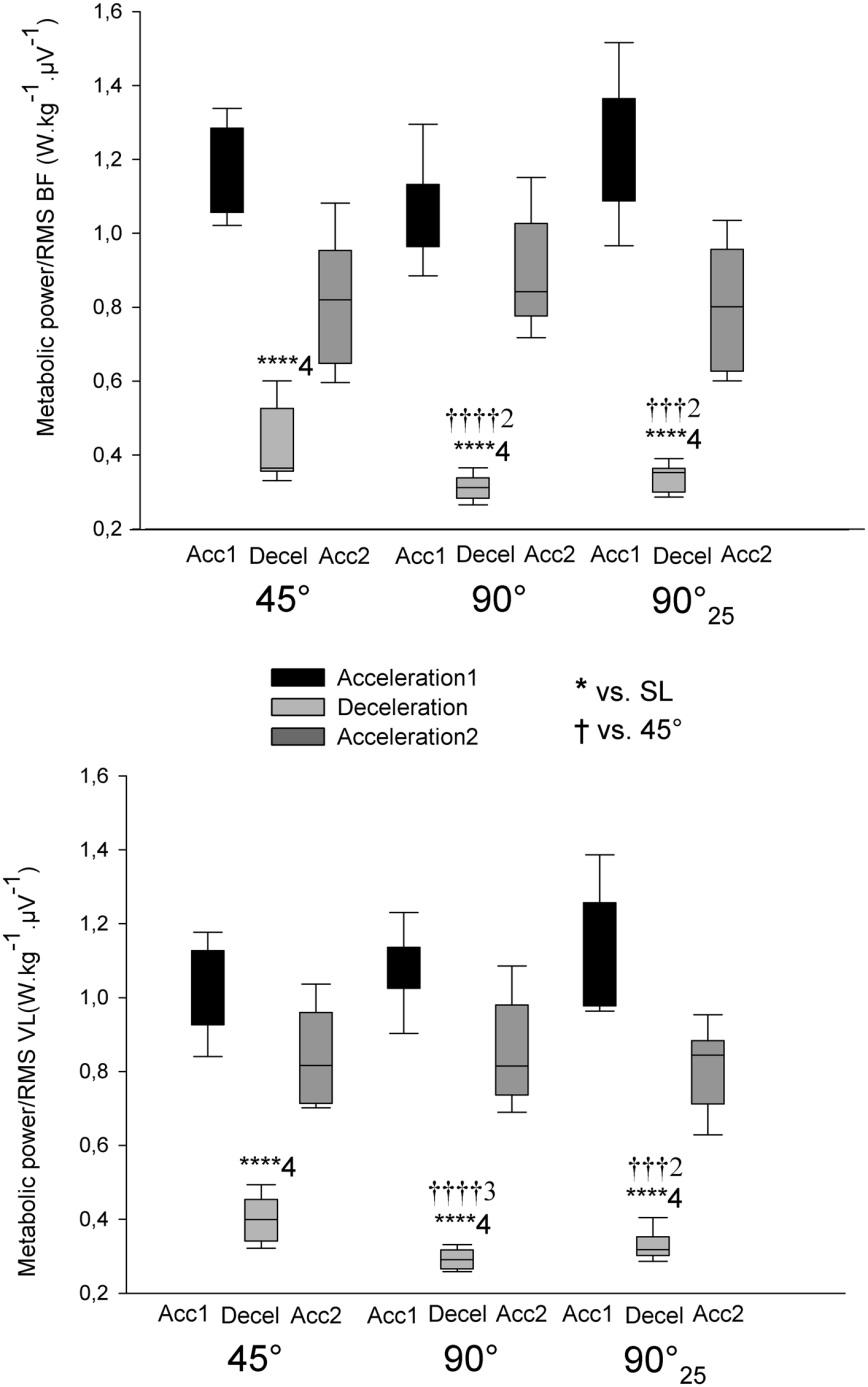
Metabolic power/electromyography amplitude (RMS) ratio during the different phases of sprints with (45° or 90°) or without (i.e., straight-line (SL)) one change of direction (COD). 90°_25_: 25-m sprint with one 90°-COD; BF: biceps femoris; VL: vastus lateralis. The number of ‘*’ and ‘†’ refers to possible, likely, very likely and almost certain difference versus straight-line and 45°-COD sprints, respectively. The associated numbers represent the magnitude of the standardized difference, with 1 standing for small, 2 for moderate, 3 for large and 4 for very large magnitude.

The overall metabolic power/RMS ratios of COD-sprints were almost certainly lower compared with straight-line (Fig 6). This ratio was angle-dependent, i.e., very likely lower with 90°-COD than with 45°-COD (Fig 6). Additionally, all deceleration phases were associated with an almost certainly lower ratio than acceleration phases and there was a very likely greater ratio during 45° than 90°-deceleration phases (Fig 7).

## Discussion

The aims of the present study were to examine both metabolic demands and lower limb muscles activity responses to field-based COD-sprints in highly-trained young soccer players. Our main findings are as follow: 1) metabolic demands were almost certainly lower during sprints with COD when compared with straight-line sprints, and this difference was even greater when accounting for the time lost when changing direction, 2) in average, VL and BF activity was slightly to almost certainly (i.e., up to ~29%) greater during sprints with COD than without, 3) the metabolic power/RMS ratio was almost certainly lower during deceleration than acceleration phase and, 4) metabolic and lower limb muscles activity demands were angle-dependent.

### Metabolic demands of COD speed

Despite the large body of research on COD speed [6], the energy expenditure of field sprinting with COD has never been reported. Similarly, the energy demands of COD- and straight-line sprints have never been compared. In the present study, we observed for the first time an almost certainly lower estimated energy expenditure during sprints with COD compared with straight-line (Fig 2). These results contrast with previous studies where changing of direction during submaximal [12, 13] and repeated sprints [10] were associated with a greater physiological load, as evidenced by increased cardiorespiratory, blood lactate and rate of perceived exertion (RPE) responses. However, while we focused in the present study on a locomotor-related indirect approach to estimate energy demands of COD-sprints, this latter was assessed in the previous studies with a direct method (e.g., systemic physiological measures such as VO_2_ and blood lactate accumulation) [10, 12, 13]. In contrast to the indirect locomotor-related approach, the direct method may take in account the greater energy demands of non-locomotor muscles involved during COD (e.g., upper-body and back muscles [10]) and the increased internal work related to the likely greater stride frequency during COD-sprints compared with uphill running [39]. While the difference in these approaches could partially explain these contrasting results, a possible underestimation of the actual metabolic cost with respect to the requirement of turning *per se*, not accounted for here, cannot be discounted [17]. An underestimation of the indirect locomotor-related approach has been also reported in three studies with differences ranging from ~15% (between 2 and 2.6 m.s^-1^, [18]) to ~30% (running at 4 m.s^-1^, [17] and during a soccer-specific circuit [40]). While two of the latter studies [17, 18] focused exclusively on 180°-CODs, the difference in the present study was ~25% with 90°-angle and ~15% with 45°-angle. The underestimation of energy demand using this approach may be related to the fact that di Prampero’s equation was established in experienced (endurance) mountain runners [31], who likely present different running economy than soccer players used in other studies [17, 18]. In fact, during uphill and downhill running (set equal to accelerated and decelerated running at an equivalent slope), the differences in energy cost may increase between experienced mountain runners and soccer players [17].

The lower locomotor-related energy expenditure during entire COD-sprints, compared with straight-line, may also be related to the very low locomotor-related metabolic demands of the deceleration phase during COD-sprints, that may not be compensated by the increased requirement of the reacceleration phase (Fig 2). In fact, the deceleration phase is characterized by both an important decrease in speed (Fig 5) and an increase in eccentric muscle contractions, which have been estimated as two to six times less metabolically demanding for the same amount of work than concentric contractions [41, 42]. Accordingly, in agreement with Osgnach et al. [7] who suggested that the metabolic demands were lower (i.e., 2.4 J.kg^-1^.m^-1^) at -2 m.s^-2^, the lowest metabolic demand (i.e., 2.6 J.kg^-1^.m^-1^) in the present study was observed when decelerating at 2.26 m.s^-2^ (Fig 3). Finally, the lower energy requirement of COD-sprint compared with straight-line speed was even more apparent when considering the sprints adjusted for the time lost when changing of direction (Fig 2), reinforcing the findings that COD *per se* may not be as metabolically demanding as we thought.

Besides the finding that COD-sprints were less metabolically demanding than in straight-line, we also found that the locomotor-related energy demands of COD-sprints were angle-dependent. Indeed, the almost certainly lower estimated energy expenditure observed during the 90°-COD compared with the 45°-COD sprints could be related to the almost certainly lower acceleration and greater deceleration distances (Table 3). These differences in acceleration and deceleration distances were associated with a likely greater peak deceleration and likely lower peak speed, peak acceleration and speed during the 90°-turn [22]. In previous studies, it was shown that the mean braking forces during the COD phase were largely greater with a 90°- than a 45°-turn at maximal running speed [25, 43]. It was concluded that greater direction change angles coincide with an increase of the mean braking forces observed during the COD phase. While a component of the initial momentum developed before the 45°-turn could be transferred to the subsequent outgoing run, the incoming momentum had to be terminated before the 90°-turn, which requires greater braking force [25, 43]. Additionally, compared with 45°-COD, largely greater re-acceleration and propelling forces associated with the 90°-COD are actually applied in the frontal plane during the impact phase [25, 44]. However, the forward acceleration is the only acceleration component used to determine the locomotor-related energy demands without taking in account the two other components [16]. Therefore, the decreased locomotor-related energy demands associated with the greater deceleration phase during the 90°-COD sprints likely explains the lower energy demands, despite the need to regenerate a new momentum for the outgoing run [25].

**Table 3:** Running variables during sprints with and without changes of direction.

	Straight-line	45°	90°	90°_25_
**Peak speed before COD (m.s**^**-1**^**)**	8.06 ± 0.46	6.65 ± 0.32 ****4	6.40 ± 0.30 ****4††2	7.11 ± 0.41 ****4††††2**ǂǂǂǂ**4
**Peak re-acceleration (m.s**^**-2**^**)**	0.98 ± 0.40	1.52 ± 0.36 ***3	2.70 ± 0.57 ****4††††3	2.66 ± 0.91 ****4††††3
**Peak deceleration (m.s**^**-2**^**)**		-1.12 ± 0.82	-3.00 ± 0.78 ††††4	-3.29 ± 0.82 ††††4
**Acceleration distance (m.s**^**-2**^**)**	20m	7.71 ± 1.74	4.41 ± 0.62 ††††4	7.55 ± 1.3 **ǂǂǂǂ**4
**Deceleration distance (m)**		4.3 ± 1.9	7.1 ± 1.2 ††††4	8.7 ± 0.9 ††††4**ǂǂǂǂ**2

COD: change of direction; SL: straight-line. The number of ‘*’, ‘†’ and ‘ǂ’ refers to possible, likely, very likely and almost certain difference versus straight-line, 45° and 90° conditions, respectively. The associated number refers to the magnitude of the difference, with 1 standing for small, 2 for moderate, 3 for large and 4 for very large magnitude.

Data from the 90°_25_ COD-sprints shows that despite a greater speed reached before the 90°_25_-COD, the peak of re-acceleration after COD was similar (Table 3). This indicates that the locomotor-related energy demands of pre-planned 90°-COD sprints may not be affected by greater acceleration and speed before COD (i.e., distance of 15 m vs. 10 m before the COD).

Finally, if we consider that the lower locomotor-related energy demands of COD sprints compared with straight-line sprints is related to very low energy demands of the deceleration phase, it can be hypothesized that sprints with greater COD angles would be associated with even lower locomotor-related energy demands.

### Lower limb muscles activity demands of COD speed

Compared with straight-line sprints, the overall lower limb muscles (i.e., VL and BF) activity was very likely-to-almost certainly greater (i.e., VL and BF) during 90°-COD sprints, and slightly greater (i.e., VL) during 45°-COD sprints (Fig 4). To our knowledge, this is the first time that lower limb muscles activity is reported during an entire sprint with COD in the field. When examining 30°- and 60°-COD submaximal runs, Besier, Lloyd (23) reported a substantial increase of VL and BF activity during the impact stance phase during (i.e., ~10 km.h^-1^), compared with straight-line. Hanson et al. [45] have shown a large increase of both VL and BF activity during the turn of a 45°-COD run. In other studies comparing 45°-COD sprints with straight line runs, muscles activity was averaged over different muscle groups (i.e., quadriceps and hamstring) and EMG activity was reported to be equivalent and lower for quadriceps and hamstring, respectively. These contradictory results may be explained by the heterogeneity of protocols and differences in study designs (e.g., EMG normalization procedure, running speed, angle of the COD, choice of lower limb muscles measured). When monitoring for the first time the muscle activity time course during each run, we found EMG activity to be phase-dependent. Compared with straight-line sprints, the magnitude of the VL and BF activity during specific portions of COD-sprints may explain the greater average VL and BF activity during the whole COD-sprints, compared with straight-line (Fig 5). COD-sprints elicited substantially greater VL and BF activities especially during and just after the turn (i.e., between the 8^th^ and the 15^th^ meter) (Fig 5). This latter portion of the sprints is generally associated with the highest deceleration (i.e., 8^th^ to 10^th^ meter) and the highest re-acceleration (i.e., 12^th^ to 15^th^ meter, Fig 5) patterns, which characterize COD-sprints with large braking and propelling forces to change of direction [25]. Present findings show that the re-acceleration effort at the end of the COD phase elicited largely (45°-COD)-to-very largely (90°-COD) greater peak accelerations compared with the second 10-m part of the straight-line sprints (Table 3). It follows from Newton's second law that greater propulsive force would be required after the turn and would explain the slightly-to-moderately (i.e., 45°-COD) and very largely (i.e., 90°-COD) greater VL and BF activities observed compared with straight-line sprints (Fig 5). In a previous study during high-intensity running, quadriceps activation increased during the impact phase of a 45°-COD and has been related to greater forces associated with COD compared with straight-line runs [46]. Quadriceps muscles are actually considered as the primary muscles to contribute to the absorption of strong eccentric forces that occur during deceleration ground contacts [37, 44]. Throughout concentric contractions, the quadriceps also help to accelerate the body in the propulsive phase of COD-runs [37]. The magnitude of the integrated hamstring activation patterns during the COD impact phase suggested that hamstring muscle group assists the knee in absorbing the forces associated with direction change [46]. In addition, the primary role of the hamstring muscles group during the COD impact phase has been identified as the mechanism by which the motion of the centre of gravity can be decelerated through the stabilisation of the knee [23, 36]. Besides the co-contraction of flexor and extensor muscle groups, a selected greater activation of BF has been also pointed out during COD to cope with internal rotation moments applied to the knee [23]. Hamstrings are also the prime movers of hip extension which is considered as a fundamental element of (horizontal) propulsion [47]. A transfer from a flexed to an extended hip angle during COD stance has been observed to coincide with high muscle activation of the hamstring group during COD tasks [46]. Therefore, the mechanical demands to change of direction while sprinting and the functional roles of VL and BF might have induced the largely greater VL and BF activities observed during the highest deceleration (8 to 11-m) and re-acceleration (11 to 15-m, Fig 5) phases of COD-sprints, compared with straight-line. During the other portions of the sprints (i.e., start to 7^th^ meter and 16^th^ to 20^th^ meter), the between-conditions difference was unclear or too small to compensate for the substantially greater VL and BF activity during the medial portion of COD-sprints (Fig 5). In addition, a recent study highlighted the magnitude of horizontal braking force during the penultimate step prior to the turn, probably as a deceleration strategy to reduce the resultant ground reaction force during last footfall and, in turn, influence knee joint loads [48].

When comparing the EMG responses to the different sprint angles over the entire runs, 90°-COD sprints elicited a possibly (i.e., BF) to very likely (i.e., VL) greater muscles activity compared with 45°-COD sprints (Fig 3). Therefore, the magnitude of difference in VL and BF activity between COD and straight-line sprints may be angle-dependent. Compared with straight-line sprints, the magnitude of the greater VL and BF activity observed during the medial portion of 45°-COD sprints was lower (i.e., small to moderate) than during the same portion of 90°-COD sprints (i.e., large to very large) (Fig 5). This difference may be associated with a largely-to-very largely greater decelerating (i.e., greater than 40%) and propelling (i.e., greater than 56%) forces required [25] to control the incoming and outgoing momentums during the 90°-COD run, respectively [25]. Accelerations tend to be greater when the movement is initiated from a standing start; in contrast, accelerations tend to be minimized when initiated once in motion. As discussed above, the lack of a need to completely regenerate a new momentum with the 45°-condition likely explains the lower reacceleration and the associated lower EMG activity compared with 90°-conditions (Table 3). It follows from Newton's second law that both the requirement for braking and propulsive forces increase with the COD angle [25].

Data from the 90°_25_ shows however that further than the angle, the actual speed reached before the COD may have only a limited effect on lower muscles activity. Actually, there was no substantial difference of BF and VL activity between 90° and 90°_25_ sprints (Fig 4). This could be explained by the similar muscles activity profile observed between conditions with an equivalent great amount of BF and VL activity during the same portion of COD sprint (i.e., just before, during and just after the turn) (Fig 5). In addition, BF and VL activities were similar during the deceleration phase (i.e., 35% and 36% of total BF and VL activities, respectively) of 90°- and 90°_25_-sprints (Fig 4). Accordingly, there was no substantial difference in peak deceleration and peak re-acceleration between 90° and 90°_25_ sprints (Table 3). This may indicate that despite largely greater peak speed and peak acceleration before the 90°_25_-COD, an equivalent pattern of VL and BF activity during deceleration and re-acceleration phases was permitted thanks to a substantially longer decelerating distance during 90°_25_ sprints (Table 3).

Overall, the present results show that a 20-m sprint with one COD may elicit a substantially greater activity demand of VL and BF than a 20-m sprint in straight-line. This difference may be explained by the mechanical demands associated with the highest deceleration and re-acceleration patterns. Additionally, VL and BF activity during COD-sprints would increase with the COD angle in relation with a greater requirement for braking and propulsive forces. Finally, the actual speed reached before the COD (during 15-m vs. 10-m) may have only a limited effect on BF and VL activity.

### Metabolic power/lower limb muscles activity ratio

There were substantial differences in the metabolic power/RMS ratio between straight-line and COD-sprints. Despite the large body of research on COD speed, the direct relation between metabolic power and muscular activity during field sprinting with COD has never been reported. The fact that COD-sprints were characterized by substantially lower metabolic power and greater RMS led to an almost certainly lower ratio compared with straight-line sprints (Fig 6). This difference highlights the dissociation between metabolic and lower limb muscular activity demands during sprints with COD, and more particularly during the deceleration phase. The metabolic power/RMS ratios of deceleration phase were 2 to 4 times (i.e., very largely) lower than that of acceleration phases (Fig 6). While the deceleration phase may be categorized by a greater lower limb muscles activity (Fig 4), the locomotor-related estimated energy expenditure is actually substantially decreased (Fig 2). It is worth noting that this dissociation between metabolic and muscular activity demands was specific to deceleration phase only, and was not observed during acceleration phases (Fig 7). In addition, this dissociation seems to be angle-dependent. Compared with the 45°-COD sprint, the metabolic power/RMS ratio was largely lower for 90°-COD sprints. This result is consistent with the substantially greater muscular activity and lower locomotor-related estimated energy expenditure found during 90°-COD sprints. The greater the COD angle, the longer the deceleration phase (Fig 5) and in turn, the lower the metabolic power (Fig 2). In addition, the greater the COD angle, the greater the braking and propelling forces [25] and the greater the lower limb muscular activity [23]. These assumptions seem to be confirmed by the largely lower 90°-COD metabolic power/RMS ratio compared with the 45°-COD sprints (Fig 6). Finally, the reported dissociation between metabolic demands and muscle activity is an additional limitation to the use of metabolic power as a single measure of running load in soccer [40].

## Conclusions

In the present study, we found that the locomotor-related metabolic demands of sprints with one COD were substantially lower than during straight-line sprints, and that this difference was even most likely greater when accounting for the time lost when changing direction. This may be related to the very low metabolic demands of the deceleration phase during COD-sprints that may not be compensated for by the increased requirement of the reacceleration phase. On the other hand, 20-m sprints with one COD may elicit a substantially greater activity of VL and BF than a 20-m sprint in straight-line. Finally, the actual speed reached before the COD may have only a limited effect on metabolic and lower limb muscular activity demands. The presence of COD also highlights the dissociation between metabolic demands and muscle activity, which directly questions the use of metabolic power as a single measure of running load in soccer.

## Supporting Information

S1 Table Estimated energy expenditure of sprints with (45° or 90°) or without one change of direction. (PDF)

S2 Table Estimated energy expenditure of sprints with change of direction-time-adjusted straight-line distances. (PDF)

S3 Table Sprints performance with (45° or 90°) or without one change of direction. (PDF)

S4 Table Change of direction-time-adjusted straight-line distances. (PDF)

S5 Table Electromyography amplitude of Vastus Lateralis muscle during sprints with (45° or 90°) one change of direction. (PDF)

S6 Table Electromyography amplitude (RMS) of Biceps Femoris muscle during sprints with (45° or 90°) one change of direction. (PDF)

S7 Table Metabolic power/electromyography amplitude (RMS) ratio of sprints with (45° or 90°) or without one change of direction. (PDF)

S8 Table Metabolic power/electromyography amplitude (RMS) ratio during the different phases of sprints with (45° or 90°) one change of direction. (PDF)
