# Supplementary material for: Metabolic Power Requirement of Change of Direction Speed in Young Soccer Players: Not All Is What It Seems
Source: PLoS One. 2016 Mar 1;11(3):e0149839. doi: 10.1371/journal.pone.0149839 (PMC4773143; doi:10.1371/journal.pone.0149839)
Supplement: S1 Table — (PDF) [file pone.0149839.s001.pdf]

S1 Table. Estimated energy expenditure of sprints with (45° or 90°) or without one change of direction

	Estimated energy expenditure ($J.kg^{-1}$)				
	SL	SL_{25}	45°	90°	$90°_{25}$
Player 1	154.2	180.0	137.0	135.7	160.2
Player 2	189.7	211.6	158.4	158.4	138.5
Player 3	146.7	171.7	132.3	139.0	154.9
Player 4	175.1	201.0	152.7	160.9	186.7
Player 5	174.0	199.4	147.3	144.8	183.3
Player 6	161.7	186.5	141.9	140.3	148.1
Player 7	169.6	195.5	148.9	124.6	189.5
Player 8	142.4	167.0	137.4	131.9	178.9
Player 9	165.1	190.3	159.8	167.7	176.1
Player 10	178.6	205.5	160.5	129.0	169.0
Player 11	172.9	195.8	154.1	138.0	189.1
Player 12	191.0	217.7	163.2	134.5	199.0

SL: straight-line; COD: change of direction; SL_{25}: 25-m straight-line sprint; 45°: 20-m sprint with one 45°-COD; 90°: 20-m sprint with one 90°-COD; $90°_{25}$: 25-m sprint with one 90°-COD
