# Supplementary material for: Metabolic Power Requirement of Change of Direction Speed in Young Soccer Players: Not All Is What It Seems
Source: PLoS One. 2016 Mar 1;11(3):e0149839. doi: 10.1371/journal.pone.0149839 (PMC4773143; doi:10.1371/journal.pone.0149839)
Supplement: S2 Table — (PDF) [file pone.0149839.s002.pdf]

S2 Table. Estimated energy expenditure of sprints with change of direction-time-adjusted straight-line distances

	Estimated energy expenditure ($J.kg^{-1}$)		
	**45°**	**90°**	**$90°_{25}$**
**Player 1**	159.5	175.0	204.1
**Player 2**	201.9	212.6	242.6
**Player 3**	166.9	166.9	195.4
**Player 4**	180.7	196.1	226.2
**Player 5**	179.2	204.1	224.5
**Player 6**	176.8	196.1	210.9
**Player 7**	185.2	200.6	220.4
**Player 8**	147.4	152.0	190.4
**Player 9**	175.3	205.1	214.9
**Player 10**	189.6	205.5	231.0
**Player 11**	186.5	195.8	220.8
**Player 12**	198.2	208.5	233.0

SL: straight-line; COD: change of direction; SL_{25}: 25-m straight-line sprint; 45°: 20-m sprint with one 45°-COD; 90°: 20-m sprint with one 90°-COD; COD-time adjusted straight-line distance: adjusted (i.e., extended) straight-line running distances matched for COD-sprint time.
