# Supplementary material for: Metabolic Power Requirement of Change of Direction Speed in Young Soccer Players: Not All Is What It Seems
Source: PLoS One. 2016 Mar 1;11(3):e0149839. doi: 10.1371/journal.pone.0149839 (PMC4773143; doi:10.1371/journal.pone.0149839)
Supplement: S3 Table — (PDF) [file pone.0149839.s003.pdf]

S3 Table. Sprints performance with (45° or 90°) or without one change of direction

	Sprint time (s)			
	**SL**	**45°**	**90°**	**$90°_{25}$**
**Player 1**	2.98	3.32	3.66	4.46
**Player 2**	2.91	3.49	3.83	4.22
**Player 3**	3.05	3.36	3.58	4.21
**Player 4**	2.88	3.20	3.65	4.16
**Player 5**	2.94	3.36	3.72	4.45
**Player 6**	2.89	3.27	3.86	4.22
**Player 7**	2.65	3.27	3.51	3.93
**Player 8**	2.98	3.48	3.79	4.45
**Player 9**	3.02	3.25	3.97	4.47
**Player 10**	2.83	3.27	3.74	4.05
**Player 11**	2.66	3.08	3.42	4.12
**Player 12**	2.90	3.26	3.69	4.24

SL: straight-line; COD: change of direction; 45°: 20-m sprint with one 45°-COD; 90°: 20-m sprint with one 90°-COD; $90°_{25}$: 25-m sprint with one 90°-COD
