# Supplementary material for: Metabolic Power Requirement of Change of Direction Speed in Young Soccer Players: Not All Is What It Seems
Source: PLoS One. 2016 Mar 1;11(3):e0149839. doi: 10.1371/journal.pone.0149839 (PMC4773143; doi:10.1371/journal.pone.0149839)
Supplement: S4 Table — (PDF) [file pone.0149839.s004.pdf]

S4 Table. Change of direction-time-adjusted straight-line distances

	Adjusted straight-line distances (m)	
	**45°**	**90°**
**Player 1**	21.4	23.7
**Player 2**	22.8	25.3
**Player 3**	24.4	24.3
**Player 4**	21.4	24.2
**Player 5**	21.2	25.5
**Player 6**	22.7	26.6
**Player 7**	23.3	25.6
**Player 8**	20.4	24.8
**Player 9**	22.3	27.9
**Player 10**	22.0	24.9
**Player 11**	22.8	25.0
**Player 12**	21.1	22.8

COD: change of direction; COD time-adjusted straight-line distance: adjusted (i.e., extended) straight-line running distances matched for change COD-sprint time; 45°: 20-m sprint with one 45°-COD; 90°: 20-m sprint with one 90°-COD
