# Supplementary material for: Metabolic Power Requirement of Change of Direction Speed in Young Soccer Players: Not All Is What It Seems
Source: PLoS One. 2016 Mar 1;11(3):e0149839. doi: 10.1371/journal.pone.0149839 (PMC4773143; doi:10.1371/journal.pone.0149839)
Supplement: S5 Table — (PDF) [file pone.0149839.s005.pdf]

S5 Table. Electromyography amplitude of Vastus Lateralis muscle during sprints with (45° or 90°) one change of direction

	Electromyographie amplitude (% RMS SL)		
	**45°**	**90°**	**$90°_{25}$**
**Player 1**	98.7	99.8	95.9
**Player 2**	107.4	113.4	126.2
**Player 3**	100.9	99.0	125.0
**Player 4**	103.1	97.5	117.0
**Player 5**	104.7	103.1	97.3
**Player 6**	106.3	115.7	109.0
**Player 7**	103.0	124.1	113.5
**Player 8**	107.2	112.2	106.7
**Player 9**	101.1	105.9	94.2
**Player 10**	98.0	95.2	100.9
**Player 11**	107.0	103.5	106.5
**Player 12**	90.4	118.3	98.0

SL: straight-line; COD: change of direction; 45°: 20-m sprint with one 45°-COD; 90°: 20-m sprint with one 90°-COD; $90°_{25}$: 25-m sprint with one 90°-COD; %RMS SL: percentage of straight-line sprint's electromyography amplitude
