# Supplementary material for: Metabolic Power Requirement of Change of Direction Speed in Young Soccer Players: Not All Is What It Seems
Source: PLoS One. 2016 Mar 1;11(3):e0149839. doi: 10.1371/journal.pone.0149839 (PMC4773143; doi:10.1371/journal.pone.0149839)
Supplement: S6 Table — (PDF) [file pone.0149839.s006.pdf]

S6 Table. Electromyography amplitude (RMS) of Biceps Femoris muscle during sprints with (45° or 90°) one change of direction

	Electromyographie amplitude (% RMS SL)		
	**45°**	**90°**	**90°$_{25}$**
**Player 1**	100.1	108.2	98.9
**Player 2**	109.7	109.7	110.7
**Player 3**	104.3	106.3	110.2
**Player 4**	95.0	105.6	109.9
**Player 5**	96.4	101.0	101.7
**Player 6**	107.5	105.5	107.9
**Player 7**	96.2	105.9	108.2
**Player 8**	102.2	105.7	110.2
**Player 9**	94.1	106.7	106.3
**Player 10**	108.6	105.5	105.0
**Player 11**	99.3	102.3	104.7
**Player 12**	107.1	109.2	97.7

SL: straight-line; COD: change of direction; 45°: 20-m sprint with one 45°-COD; 90°: 20-m sprint with one 90°-COD; 90°$_{25}$: 25-m sprint with one 90°-COD; %RMS SL: percentage of straight-line sprint's electromyography amplitude
