# Supplementary material for: Metabolic Power Requirement of Change of Direction Speed in Young Soccer Players: Not All Is What It Seems
Source: PLoS One. 2016 Mar 1;11(3):e0149839. doi: 10.1371/journal.pone.0149839 (PMC4773143; doi:10.1371/journal.pone.0149839)
Supplement: S7 Table — (PDF) [file pone.0149839.s007.pdf]

**S7 Table. Metabolic power/electromyography amplitude (RMS) ratio of sprints with (45° or 90°) or without one change of direction**

| | Biceps Femoris Metabolic power/RMS ratio ($W.kg^{-1}.\mu V^{-1}$) | | | |
|---|---|---|---|---|
| | **SL** | **45°** | **90°** | **$90°_{25}$** |
| **Player 1** | 1.01 | 0.68 | 0.61 | 0.64 |
| **Player 2** | 0.99 | 0.77 | 0.76 | 0.55 |
| **Player 3** | 0.99 | 0.60 | 0.65 | 0.65 |
| **Player 4** | 0.99 | 0.97 | 0.81 | 0.79 |
| **Player 5** | 1.00 | 0.91 | 0.66 | 0.75 |
| **Player 6** | 1.01 | 0.69 | 0.74 | 0.61 |
| **Player 7** | 0.96 | 0.84 | 0.64 | 0.77 |
| **Player 8** | 1.01 | 0.84 | 0.65 | 0.70 |
| **Player 9** | 1.02 | 0.86 | 0.85 | 0.81 |
| **Player 10** | 1.01 | 0.73 | 0.68 | 0.72 |
| **Player 11** | 0.95 | 0.94 | 0.73 | 0.78 |
| **Player 12** | 1.03 | 0.81 | 0.70 | 0.84 |

| | Vastus Lateralis Metabolic power/RMS ratio ($W.kg^{-1}.\mu V^{-1}$) | | | |
|---|---|---|---|---|
| | **SL** | **45°** | **90°** | **$90°_{25}$** |
| **Player 1** | 0.99 | 0.73 | 0.67 | 0.75 |
| **Player 2** | 0.99 | 0.77 | 0.72 | 0.58 |
| **Player 3** | 0.98 | 0.80 | 0.67 | 0.62 |
| **Player 4** | 1.02 | 0.78 | 0.79 | 0.90 |
| **Player 5** | 1.03 | 0.82 | 0.70 | 0.76 |
| **Player 6** | 1.00 | 0.73 | 0.80 | 0.59 |
| **Player 7** | 1.02 | 0.77 | 0.61 | 0.70 |
| **Player 8** | 1.02 | 0.83 | 0.67 | 0.75 |
| **Player 9** | 0.96 | 0.88 | 0.75 | 0.78 |
| **Player 10** | 1.01 | 0.92 | 0.68 | 0.82 |
| **Player 11** | 0.98 | 0.83 | 0.74 | 0.74 |
| **Player 12** | 0.97 | 0.91 | 0.67 | 0.81 |

SL: straight-line; COD: change of direction; 45°: 20-m sprint with one 45°-COD; 90°: 20-m sprint with one 90°-COD; $90°_{25}$: 25-m sprint with one 90°-COD; Upper and lower panels concerning Biceps Femoris and Vastus Lateralis muscles, respectively.
