# Supplementary material for: Metabolic Power Requirement of Change of Direction Speed in Young Soccer Players: Not All Is What It Seems
Source: PLoS One. 2016 Mar 1;11(3):e0149839. doi: 10.1371/journal.pone.0149839 (PMC4773143; doi:10.1371/journal.pone.0149839)
Supplement: S8 Table — (PDF) [file pone.0149839.s008.pdf]

S8 Table. Metabolic power/electromyography amplitude (RMS) ratio during the different phases of sprints with (45° or 90°) one change of direction

Biceps Femoris	Metabolic power/RMS ratio								
	Acceleration 1			Deceleration			Acceleration 2		
	45°	90°	$90°_{25}$	45°	90°	$90°_{25}$	45°	90°	$90°_{25}$
Player 1	1.09	1.01	1.19	0.34	0.29	0.32	0.68	0.72	0.61
Player 2	1.07	0.98	0.93	0.43	0.31	0.29	0.64	1.17	0.60
Player 3	1.07	1.10	1.57	0.40	0.27	0.29	0.58	0.72	0.67
Player 4	1.06	1.03	1.39	0.62	0.37	0.36	1.13	1.05	0.90
Player 5	1.01	0.95	1.38	0.52	0.29	0.36	0.98	0.85	0.80
Player 6	1.05	1.14	1.04	0.37	0.36	0.34	0.80	0.78	0.61
Player 7	1.33	0.86	1.20	0.36	0.32	0.37	0.94	0.98	0.97
Player 8	1.29	1.12	1.09	0.36	0.28	0.30	0.77	0.78	0.92
Player 9	1.04	0.96	1.09	0.53	0.32	0.39	0.93	1.12	1.06
Player 10	1.26	1.09	1.30	0.33	0.32	0.35	0.63	0.83	0.69
Player 11	1.26	1.20	1.33	0.53	0.35	0.36	0.96	0.89	0.80
Player 12	1.34	1.34	1.21	0.36	0.27	0.39	0.84	0.83	0.98

Vastus Lateralis	Metabolic power/RMS ratio								
	Acceleration 1			Deceleration			Acceleration 2		
	45°	90°	$90°_{25}$	45°	90°	$90°_{25}$	45°	90°	$90°_{25}$
Player 1	1.10	1.03	1.25	0.34	0.29	0.35	0.72	0.81	0.72
Player 2	0.84	1.03	0.97	0.41	0.27	0.29	0.72	1.00	0.62
Player 3	1.03	0.91	0.96	0.46	0.31	0.30	0.77	0.74	0.72
Player 4	0.92	1.13	1.41	0.35	0.34	0.43	0.88	1.04	0.96
Player 5	0.84	1.02	1.33	0.44	0.30	0.35	0.90	0.83	0.78
Player 6	0.99	1.06	0.96	0.35	0.34	0.32	0.71	0.76	0.67
Player 7	0.95	0.90	1.01	0.33	0.27	0.31	0.91	0.93	0.89
Player 8	1.15	1.14	1.17	0.38	0.30	0.31	0.75	0.71	0.89
Player 9	0.94	1.13	1.24	0.46	0.27	0.33	1.02	1.12	0.89
Player 10	1.09	1.10	1.26	0.46	0.30	0.38	0.99	0.82	0.87
Player 11	1.14	1.24	1.14	0.51	0.33	0.31	0.71	0.85	0.83
Player 12	1.19	1.21	1.07	0.41	0.28	0.36	1.05	0.69	0.96

SL: straight-line; COD: change of direction; 45°: 20-m sprint with one 45°-COD; 90°: 20-m sprint with one 90°-COD; $90°_{25}$: 25-m sprint with one 90°-COD; Upper and lower panels concerning Biceps Femoris and Vastus Lateralis muscles, respectively.
